# Low‐calorie functional dairy dessert enriched by prebiotic fibers and high antioxidant herbal extracts: A study of optimization and rheological properties

**DOI:** 10.1002/fsn3.4189

**Published:** 2024-08-13

**Authors:** Seyyedeh Leila Hosseinipour, Babak Ghanbarzadeh, Vahid Mofid, Mostafa Soltani, Hedayat Hosseini

**Affiliations:** ^1^ Department of Food Sciences and Technology, Faculty of Pharmacy, Tehran Medical Sciences Islamic Azad University Tehran Iran; ^2^ Department of Food Science and Technology, Faculty of Agriculture University of Tabriz Tabriz Iran; ^3^ Department of Food Science and Technology, National Nutrition and Food Technology Research Institute, Faculty of Nutrition Science and Food Technology Shahid Beheshti University of Medical Sciences Tehran Iran

**Keywords:** antioxidant, functional dessert, inulin, optimization, polydextrose, rheology

## Abstract

The formulations of functional low‐calorie dairy dessert, enriched with inulin/polydextrose (as a starch substitute), and ginger/cinnamon extract (as a flavor component and natural antioxidants), were developed and optimized by the D‐optimal mixed design method. In the first stage, using the hedonic sensory evaluation and syneresis data, the optimal concentrations of inulin and polydextrose were obtained as 2.49% inulin and 1.51% polydextrose, respectively. The steady shear rheological test showed that the replacement of starch with inulin and polydextrose caused a decrease in apparent viscosity in all dessert samples. This decrease was higher in the samples containing polydextrose than those containing inulin. The replacement of starch with inulin and polydextrose also reduced the hysteresis loop area and thixotropic behavior. In the second stage, 0–0.4% of ginger and cinnamon extracts  were added to the optimum sample and then the antioxidant and color properties of dessert samples were evaluated. The lightness (L*) Hunter parameter decreased by adding extracts and the samples containing cinnamon extract showed a higher a* parameter than the control and ginger‐incorporated samples. The result of the 2,2‐diphenyl 1‐picrylhydrazyl (DPPH) antioxidant assay showed that the antioxidant capacity of ginger extract was significantly higher than that of cinnamon extract. The half‐maximal inhibitory concentration (IC50) value of dessert samples decreased by adding 0.4% cinnamon and ginger extracts from 88.30 mg/mL to 77.04 and 31.94 mg/mL, respectively.

## Graphical abstract

The steady shear rheological test showed that the replacement of starch with inulin and polydextrose caused a decrease in apparent viscosity, hysteresis loop area, and thixotropic behavior in all functional low‐calorie dessert samples and this decrease was higher in the samples containing polydextrose than those containing inulin. The optimal concentrations of inulin and polydextrose were obtained as 2.49% inulin and 1.51% polydextrose, respectively, on the basis of sensory and synersis tests. The result of the 2,2‐diphenyl 1‐picrylhydrazyl (DPPH) antioxidant assay showed that the antioxidant capacity of the sample containing ginger extract was significantly higher than that of cinnamon extract.

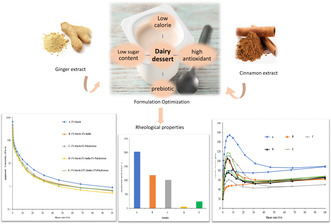

## 1 INTRODUCTION

Nowadays, diabetes is considered as one of the most important and serious chronic diseases. Its global spread has reached epidemic proportions and it is predicted that the number of people suffering from this disease will increase to about 783 million people in the world by 2045. One of the causes of diabetes is obesity and consumption of high‐calorie foods, hence the demand for production and consuming low glycemic index foods will increase (Sun et al., 2022). Today, using new technologies in the food industry facilitates produce of food products with more health benefits, such as functional foods. In the development of healthy beneficial food products, the choice of initial food matrix is very important. Dairy desserts seem to be a good choice for the development of healthy foods because they are widely consumed around the world by different age groups. On the other hand, the consumption of these products is directly related to the sensory and nutritional properties of desserts (Farias et al., 2019; Osborn & Sinn, 2007). It is becoming increasingly clear that the overconsumption of foods and beverages high in sugar plays an important role in the increase of overweight, obesity, and chronic diseases like diabetes, metabolic syndrome, cardiovascular disease, and hypertension. Replacing sugar with low‐calorie/no‐calorie high‐intensity sweeteners can be potentially a good solution for decreasing these disorders. Sucralose, stevia, saccharin, aspartame, acesulfame potassium, and neotame are the most important high‐intensity sweeteners. The biggest disadvantage of these sweeteners is that, unlike sugars, they cannot create a proper texture and consistency in foods, and their combination with alcoholic sugars, such as sorbitol, can be a good solution to this problem.

Among the prebiotic and dietary fiber compounds, inulin and polydextrose are known as one of the most important functional food ingredients, both of which are indigestible polysaccharides and soluble dietary fibers (Fakhri et al., 2023). Their limited digestibility in the small intestine and their fermentation in the colon induce a preventive effect on the growth of pathogens and harmful microorganisms and also increase the feeling of satiety (Gibson et al., 2017). On the other hand, these compounds have technological advantages and they can act as substitutes for fat or sugar in food formulations, and they affect the physicochemical, rheological, organoleptic, and textural properties of the final product (Costa et al., 2019).

Ginger and cinnamon extracts have unique tastes and antioxidant properties that are extensively used in food formulations. The antioxidant properties of ginger are related to phenolic phytochemical compounds, such as gingerol, shogaol, and hexahydrocurcumin. Shogoal is the main compound responsible for the pungent taste of ginger, which also has heart‐protective properties due to its anticoagulant properties. The fresh ginger rhizome contains gingerol, which after drying converts into zingerone, shogoal, etc., with different levels of antioxidant and anti‐inflammatory properties (Bandyopadhyay et al., 2008).

Cinnamon bark extract has a wide range of volatile compounds, such as cinnamaldehyde, eugenol, linalool, and limonene. Phytochemicals derived from cinnamon have been widely used in traditional medicine around the world since ancient times and are still of medical importance today. Cinnamon is widely used in traditional foods and drinks due to its unique flavor, and recently, its antimicrobial and antioxidant properties have been used to treat influenza, inhibit the proliferation of cancer cells, protect against heart disease, reduce inflammation, and improve glucose metabolism. It has been stated that cinnamon extract can potentiate insulin action and insulin receptor function (Abdelwahab et al., 2017).

According to our knowledge, the combined effect of prebiotic (inulin and polydextrose), and sucralose–sorbitol low‐calorie sweetener on the sensory and rheological properties of dairy desserts have not been investigated.

This research work aimed to produce functional low‐calorie dairy desserts with optimal sensory and rheological properties, which have three favorable and health‐giving properties, including having low calories, high fiber and prebiotics, and high antioxidant properties. For optimization and arriving at this aim, sucralose–sorbitol sweeteners, inulin and polydextrose prebiotic fibers, and cinnamon and ginger extracts were used in the formulation based on the mixed design method.

## 2 MATERIALS AND METHODS

### 2.1 Materials

Sorbitol powder was from Zhejiang and sucralose from JK Sucralose Inc., China, long‐chain inulin (degree of polymerization (DP) ≥23) from BENEO, Belgium, polydextrose (E 1200) from Fooding, China, ginger and cinnamon extracts from Naturex, France, and skim milk, skim milk powder, starch, cream (40% fat), κ‐carrageenan, fructose syrup, and sugar were all obtained from Domino's Dairy Products Company in Tehran.

### 2.2 Preparing low‐calorie dairy dessert

To prepare the dessert, first, all the fixed powder components including non‐fat dry milk (2%), κ‐carrageenan (0.5%), sorbitol (13%), sucralose (0.05%), and variable powder components including starch, inulin, and polydextrose were mixed according to the percentages mentioned in Table 1. Then using a Thermomix food processor (TN 31, Wuppertal, Germany), the powder components were dissolved in skim milk (69.5%) and cream (10%), at 10°C for 5 min, and to have a homogeneous composition, the dessert was stirred for 30 min at 350 rpm (revolutions per minute) at 45°C. Afterward, pasteurization was carried out at 73°C for 30 s, the dessert was then poured (hot‐filling) into 100 cc plastic containers, and stored at 4°C for further experiments. Based on the results of experiments, the optimal low‐calorie dessert formulation was determined in terms of prebiotics concentration with the help of Design Expert software. In the second step, after adding different concentrations of 0–0.4% of ginger and cinnamon extracts to the optimized sample, their color and antioxidant properties were checked (Table 7). Finally, the optimized dessert with the highest antioxidant properties was selected as the final product.

**TABLE 1:** The experimental design and results of D‐optimal mixture experiment.

Run	X_1_	X_2_	X_3_	Y_1_	Y_2_	Y_3_	Y_4_	Y_5_	Y_6_
	Starch %	Inulin %	PD %	Syneresis %	Texture	Taste	Aroma	Appearance	Overall
1	1	0	4	2.9	7.7	6.14	5.32	7.78	6.88
2	0	2.5	2.5	4.4	4.1	3.84	3.44	4.08	3.82
3	0.5	4.5	0	3.2	7.1	5.5	7.5	5.44	5.94
4	1	4	0	2.5	8.1	7.42	7.14	7.64	7.88
5	0	5	0	4.1	4.3	4.14	4.66	3.9	4.54
6	0.75	1.125	3.125	3	7.5	5.58	5.62	6.7	6
7	0	2.5	2.5	4.5	3.9	3.84	4.5	4.08	3.82
8	0	5	0	4.2	3.7	4.14	4.66	3.9	4.54
9	1	0	4	2.8	7.9	6.14	5.32	7.78	6.88
10	1	2	2	2.7	7.9	6.88	6.06	8.04	7.64
11	1	4	0	2.4	8.3	7.42	7.14	7.64	8.18
12	0	0	5	4.9	3.7	3.18	2.74	3.26	2.78
13	0.25	3.625	1.125	3.6	6.5	4.62	5.28	4.8	4.84
14	0	0	5	5.02	3.3	3.18	2.74	3.26	2.78
15	0.75	3.125	1.125	3.1	7.1	6.16	6.5	6.72	6.68
16	0.5	0	4.5	3.4	6.9	3.7	4.84	5.7	5.1

### 2.3 Sensory evaluation

A sensory evaluation of the samples was performed to determine the optimal formulation (replacement ratio of inulin and polydextrose as a starch substitute). For this purpose, 34 semi‐trained panelists aged 28 to 52 years, including 9 men and 5 women drawn from Domino's Dairy Products Company, participated in the evaluation. The samples were randomly coded with three‐digit numbers and were kept outside the refrigerator for 1 h before the test to reach the ambient temperature and were given to the panelists along with a glass of water for mouthwash. A 9‐point hedonic scale ranging from 1 (dislike extremely) to 9 (like extremely) was used to measure acceptance level. All samples were evaluated for sensory attributes, including taste, aroma, texture, appearance, and overall acceptance (Bhalerao et al., 2020; Samakradhamrongthai et al., 2021). The selection of attributes was done based on the agreement between the evaluators and the vocabulary of each attribute is in line with the International Organization for Standardization No. 5492 (ISO 2008).

### 2.4 Physicochemical analyses

#### 2.4.1 Syneresis measurement

Syneresis of the samples was evaluated in triplicate using a centrifuge model D‐6360 made in Germany. First, 20 mL of freshly prepared desserts was placed in 50 mL tubes and stored at 4°C for 4 days. Then each sample was centrifuged at 2000 *g* for 30 min. The obtained clear liquid at the top of the tubes was poured off, and then weighed. Syneresis was expressed as a percentage of the weight of the released liquid compared to the initial weight of the dessert (Li et al., 2019).

#### 2.4.2 Color measurement

The colorimetric test was performed in two stages to investigate the effect of prebiotics and extracts on the color change of the dessert. Color measurement was performed after storage of the samples until reaching the ambient temperature using the HunterLab EZ 45.0 LAZ ColorFlex colorimeter made in the United States of America. The color of the samples was measured based on L* (lightness corresponding to light and dark), a* (positive values indicate red and negative values indicate green color), b* (positive values indicate yellow and negative values indicate blue color), and ∆*E* (total color change calculated according to Equation (1) values in four different parts by placing a flat part of the sample on the white standard tile in three repetitions (Kuriya et al., 2020)).

(1) $$∆E=\sqrt{{\left(L_{s}^{*}-L_{c}^{*}\right)}^{2}+{\left(a_{s}^{*}-a_{c}^{*}\right)}^{2}+{\left(b_{s}^{*}-b_{c}^{*}\right)}^{2}}$$

#### 2.4.3 Antioxidant capacity (2,2 diphenyl‐1‐picrylhydrazyl assay)

To determine the antioxidant properties of ginger and cinnamon extracts in the dessert formulation, the method of Khodaei et al. (2021) was used with modifications to determine the radical activity of 2,2‐diphenyl‐1‐picrylhydrazyl (DPPH). For this purpose, 1 g of each sample was taken and 2 mL of 99.5% methanol was added to it and mixed well. After 20 min, the samples were centrifuged at 10 000 rpm for 15 min and the dessert extract was obtained. Then 150 μL of the methanol solution of the desired sample was poured into a test tube containing 2 mL of 0.1 mM DPPH solution. The contents of each tube were completely vortexed and after 30 min, at room temperature and in the dark, the absorbance of the samples was read at 517 nm wavelength with an Ultraviolet/Visible (UV/Vis) spectrophotometer (UNICO, USA). The inhibition percentage of DPPH scavenging was calculated as follows and the concentration of samples required to inhibit 50% of primary DPPH radicals was expressed as half‐maximal inhibitory concentration (IC50).

$$\text{Inhibition percentage}\;\left(\%I\right)=\left(\mathrm{Abs}\;\text{initial}-\mathrm{Abs}\;\text{sample}/\mathrm{Abs}\;\text{initial}\right)\times 100$$

### 2.5 Rheological measurements

#### 2.5.1 Steady shear flow test

To determine the rheological properties of desserts, five samples, including sample A (control): 5% Starch, sample B: 1% Starch and 4% Inulin, sample C: 1% Starch and 4% Polydextrose (PD), sample D: 1% Starch, 2% Inulin, and 2% Polydextrose, and sample E (optimal sample): 1% Starch, 2.5% Inulin, and 1.5% Polydextrose, were selected. The rheological analyses of samples were performed in an Anton Paar rheometer (Anton Paar GmbH, MRC 301, Australia), using parallel‐plate geometry (50 mm diameter, 0.05 mm gap). Viscosity curves were determined at 25°C at an increasing shear rate from 0.1 to 100 s^−1^ (Gałkowska et al., 2020).

#### 2.5.2 Thixotropic behavior evaluation

Shear stress was measured as a function of increasing shear rate from 0.1 to 100 s^−1^ (upward curve), holding at 100 s^−1^ for 20 s, then decreasing from 100 s^−1^ to 0.1 s^−1^ (downward curve). The area between up–down curves of the shear stress, which is called hysteresis, was calculated using Rheoplus/32 software (version V3.40) (Arcia et al., 2010).

#### 2.5.3 Dynamic oscillatory shear (viscoelastic) tests

The linear viscoelastic region (LVR) was determined through dynamic strain sweeps ranging from 0.01% to 100% at a fixed angular frequency of 1 Hz and 25°C. Frequency sweep tests were then performed in the frequency range of 0.1–50 Hz at a constant strain of 1% (LVR) and 25°C. Viscoelastic parameters, such as storage modulus (G'), loss modulus (G″), loss tangent (tan *δ*), and complex viscosity (η*), were used for rheological evaluation of functional desserts (Arancibia et al., 2015).

### 2.6 Experimental design and statistical analysis

Design Expert software (7.1.5) was used to establish the optimal concentration of the greatest formulation. In this study to optimize and construct a reliable model, the D‐optimal mixed design method with three factors and two levels was used. The selected independent variables were Starch (X1), Inulin (X2), and Polydextrose (X3). Responses (dependent variables) included Syneresis (Y1) and sensory evaluation results, Texture (Y2), Taste (Y3), Aroma (Y4), Appearance (Y5), and Overall acceptance (Y6). The concentrations of starch, inulin, and polydextrose were considered as 0–1%. 0–5%, and 0–5%, respectively (sum of concentrations of three components was 5%) in the design of the experiment and 16 formulations (runs) were suggested by the Design Expert software (Table 1). To verify the differences between the means, SPSS software was used with Duncan's multirange test at a significance level of *p* ≤ .05, and to draw graphs, Excel 2019 software was applied.

## 3 RESULTS AND DISCUSSION

### 3.1 Analysis of D‐optimal mixture experiments, optimization, and model validation

The number of 16 formulations suggested by the Design Expert software was prepared and experiments were conducted on them. The list of dependent and independent variables and the results of each experiment are listed in Table 1. After analyzing the syneresis and sensory results, the appropriate regression models are fitted. The quadratic model was statistically significant for all responses (*p* < .0001). The Lack of Fit parameter was non‐significant (*p* > .05) (Table 2) and implying that the major part of the residual error is related to the pure error and does not relate to the adequacy of the selected model. According to the obtained coefficient of determination (*R* ^2^) for Syneresis (Y_1_), Texture (Y_2_), Taste (Y_3_), Aroma (Y_4_), Appearance (Y_5_), and Overall acceptance (Y_6_), 0.99, 0.97, 0.99, 0.96, 0.99, and 0.97, respectively, indicating the quadratic mathematical model with three independent variables did not account for only a small amount of changes in dependent variables and on the other world, over 96% of the changes in the dependent variables can be described by three independent variables. The linear effects of starch, inulin, and polydextrose concentrations on different sensory (Y1–Y5) parameters and Syneresis (Y6) were significant. The interaction effects of starch–inulin and starch–polydextrose are also significant on dependent variables (*p* < .05).

**TABLE 2:** Analysis of variance (ANOVA) in a mixed design for different desserts.

	Sum of squares	df	Mean square	*F*‐value	*p*‐value	Sum of squares	df	Mean square	*F*‐value	*p*‐value
Source	Y1: syneresis					Y2: texture				
Model	10.66	5	2.13	799.30	<.0001	52.65	5	10.53	74.11	<.0001
Linear mixture	10.46	2	5.23	1960.68	<.0001	49.76	2	24.88	175.10	<.0001
AB	0.12	1	0.12	44.74	<.0001	2.74	1	2.74	19.30	.0014
AC	0.14	1	0.14	52.49	<.0001	2.81	1	2.81	19.74	.0012
BC	9.29	1	9.293E‐003	3.48	.091	0.021	1	0.021	0.15	.7085
Residual	0.027	10	2.667E‐003			1.42	10	0.14		
Lack of fit			9.085E‐003	0.64	.68	1.10	5	0.22	3.44	.1006
*R* ^2^	.997					.937				.937
	Y3: taste					Y4: Aroma				
Model	31.95	5	6.39	272.42	<.0001	30.60	5	6.12	48.78	<.0001
Linear mixture	31.02	2	15.51	661.18	<.0001	28.38	2	14.19	113.10	<.0001
AB	0.58	1	0.58	24.58	.0006	2.19	1	2.19	17.75	.0019
AC	0.67	1	0.67	28.36	.0003	2.10	1	2.10	16.70	.002
BC	0.16	1	0.16	6.67	.0273	7.632E‐003	1	9.293E‐003	0.061	.81
Residual	0.23	10	0.023			1.25	10	0.13		
Lack of fit	0.23	5	0.047			0.69	5	0.14	1.23	.411
*R* ^2^	.992					.96				
	Y5: Appearance					Y6: overall				
Model	48.06	5	9.61	726.85	<.0001	44.81	5	8.96	94.68	<.0001
Linear mixture	47.33	2	23.66	1789.37	<.0001	43.87	2	21.93	231.73	<.0001
AB	0.095	1	0.095	7.15	.0234	0.75	1	0.75	7.96	.018
AC	0.058	1	0.058	4.40	.0623	0.69	1	0.69	7.31	.022
BC	0.39	1	0.39	29.53	.0003	0.18	1	0.18	1.92	.196
Residual	0.13	10	0.013			0.95	10	0.095		
Lack of fit	0.13	5	0.026			0.95	5	0.19		
*R* ^2^	.997					.979				

After the removal of non‐significant regression coefficients, they were simplified to the following equations.

(2) $$\text{Y1}=2.72\;X_{1}+0.83\;X_{2}+0.97\;X_{3}+0.87\;X_{1}\;X_{2}+0.94\;X_{1}\;X_{3}$$

(3) $$\text{Y2}=12.05\;X_{1}+0.82\;X_{2}+0.71\;X_{3}+4.19\;X_{1}\;X_{2}+4.23\;X_{1}\;X_{3}$$

(4) $$\text{Y3}=11.84\;X_{1}+0.83\;X_{2}+0.61\;X_{3}+1.92\;X_{1}\;X_{2}+2.06\;X_{1}\;X_{3}+0.043\;X_{2}\;X_{3}$$

(5) $$\text{Y4}=11.66\;X_{1}+0.96\;X_{2}+0.55\;X_{3}+3.74\;X_{1}\;X_{2}+3.65\;X_{1}\;X_{3}$$

(6) $$\text{Y5}=7.6\;X_{1}+0.77\;X_{2}+0.66\;X_{3}+0.77\;X_{1}\;X_{2}+0.6\;X_{1}\;X_{3}$$

(7) $$\text{Y6}=13.16\;X_{1}+0.86\;X_{2}+0.57\;X_{3}+2.19\;X_{1}\;X_{2}+2.10\;X_{1}\;X_{3}$$

**FIGURE 1:**
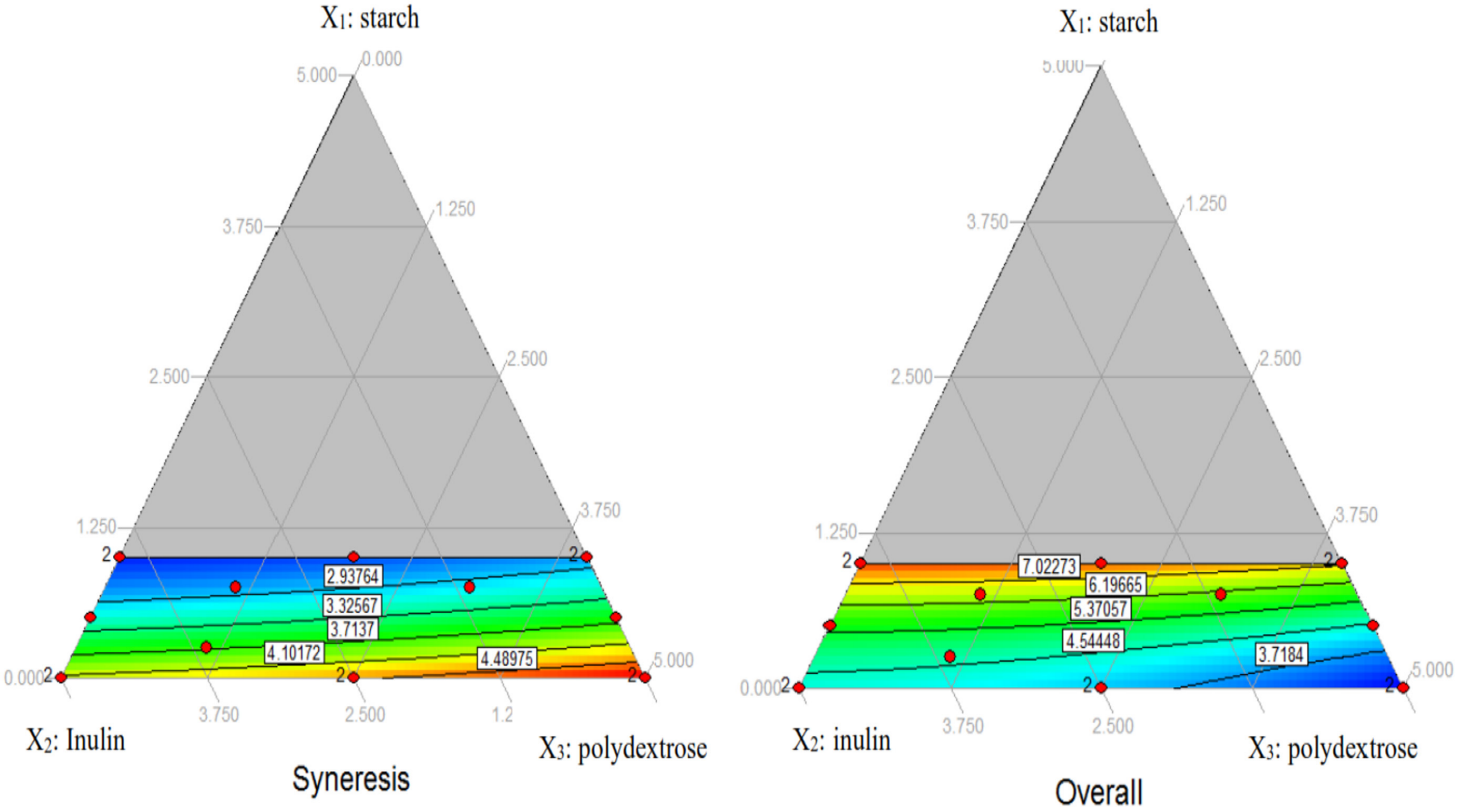
Triangular contour plot illustrating the effect of Starch (X_1_), Inulin (X_2_), and Polydextrose (X_3_) on Syneresis (Y1) and Overall acceptance (Y6).

The effect of the independent variables on the syneresis and overall acceptance in the sensory evaluation can be explained with the help of the triangular contour plot. As it can be seen in Figure 1, by increasing the concentration of starch, the syneresis decreased, and on the contrary, by increasing the concentration of inulin and polydextrose, the syneresis increased. The highest syneresis was related to the lowest percentage of starch and the highest percentage of polydextrose. Also, in high concentrations, polydextrose has a more negative effect on syneresis than inulin. The triangular contour diagrams obtained from the results of the sensory evaluation were similar to each other panelist's scores decreased with the increase in the concentration of inulin and polydextrose from low to high levels.

In the following, optimization was done to arrive at the minimum syneresis and the highest sensory evaluation score, so that the concentration of starch is within the target range of 0–1% and the concentrations of inulin and polydextrose are within the maximum range of 0–5%. All factors and responses with their restrictions are shown in Table 3. The optimal formula obtained by the software was 1% starch, 2.49% inulin, and 1.51% polydextrose. To validate the adequacy of the model equations, verification experiments were conducted with the optimal formulation suggested by the software. The experimental and predicted data for the responses (sensory and syneresis test results) at optimum points were compared using the paired t‐test method. There were no significant differences between the observed and predicted values (Table 4). Therefore, verification experiments demonstrated the adequacy of the response surface equations for responses and the optimal formulation suggested by the software was acceptable and could be used as an alternative formulation for the preparation of low‐calorie dairy desserts containing starch.

**TABLE 3:** The target range of the test factors and the response value.

Name	Goal	Lower limit	Upper limit
X_1_: Starch %	Is in range	0	1
X_2_: Inulin %	Maximize	0	5
X_3_: PD %	Maximize	0	5
Y_1_: Syneresis %	Minimize	2.5	4.89
Y_2_: Texture	Maximize	3.3	8.3
Y_3_: Taste	Maximize	3.18	7.42
Y_4_: Aroma	Maximize	2.74	7.5
Y_5_: Appearance	Maximize	3.26	8.04
Y_6_: Overall	Maximize	2.86	7.98

**TABLE 4:** Paired t‐test to compare differences between the actual and predicted value of the optimized formula.

Parameter	Predicted value	Actual value	*p*‐value
Syneresis	2.66 ± 0.061	3.49 ± 0.08	.662
Texture	7.98 ± 0.02	7.92 ± 0.27	.736
Taste	7.11 ± 0.041	7.06 ± 0.221	.746
Aroma	6.38 ± 0.19	6.25 ± 0.37	.459
Appearance	7.93 ± 0.062	7.65 ± 0.350	.331
Overall	7.73 ± 0.17	7.72 ± 0.52	.99

*Note*: Significant at *p*‐level < .05.

### 3.2 Color measurement

Although the effect of prebiotics could not be detected with the naked eye and all the samples looked white, the results of the colorimeter showed that the Hunter color values L*, a*, and b* varied from 87.51 to 88.52, −1.8 to −2.17, and 13.93 to 14.38, respectively. Most of the changes were related to value a*, which indicates an increase in the greenness of the samples with the replacement of starch with the use of prebiotics. The greenness degree is higher in samples containing polydextrose compared to the control sample and the sample containing inulin. As shown in Table 5, considering that different letters indicate a significant difference (*p* < .05), the optimized sample had a significant difference in all colorimetric values except b* compared to the control sample. The use of inulin and polydextrose has reduced the brightness of L* compared to the control sample, and the effect of polydextrose in reducing L* has been even greater. This result is in agreement with the Samakradhamrongthai et al.'s (2021) research, where the use of inulin in the optimization of low‐fat ice cream was investigated using the response surface methodology (RSM). Since the value b* depends on the amount of carotenoid in milk fat, due to no change in fat content in the formulation of desserts in this research, no significant change in the degree of yellowness has been observed (Samakradhamrongthai et al., 2021). The different results obtained in this research can be explained by the higher concentrations of inulin and polydextrose, the interaction between each other, and other ingredients in this matrix (Li et al., 2019; Naeli et al., 2022).

**TABLE 5:** Effect of replacing starch with prebiotics on the color parameter of dessert.

Sample	Formulation	L*	a*	b*	∆E*
1	5% Starch	89.38 ± 0.2^a^	−1.8 ± 0.11‑	14.32 ± 0.22^a^	0.12 ± 0.1^d^
2	1% Starch–4% Inulin	88.52 ± 0.03^b^	−2.01 ± 0.02^b^	14.28 ± 0.06^a^	0.9 ± 0.03^c^
3	1% Starch–4% PD	87.51 ± 0.01^c^	−2.15 ± 0.05^c^	14.38 ± 0.17^a^	1.89 ± 0.02^a^
4	1% Starch–2% Inulin–2% PD	88.16 ± 0.047^c^	−2.17 ± 0.05^c^	13.93 ± 0.2^b^	1.32 ± 0.1^b^
5	1% Starch–2.5% Inulin–1.5% PD	88.04 ± 0.1^d^	−2.09 ± 0.04^c^	14.13 ± 0.03^ab^	1.41 ± 0.11^b^

*Note*: Different letters denote significant differences (*p* < .05).

As shown in Table 6, the effect of using ginger and cinnamon extracts on all samples was significantly different from the control sample. Although in the samples with both extracts, the color difference of the desserts was not visible to the naked eye, in the sample containing ginger and cinnamon extracts individually, the L* values were 84.52 and 66.81, respectively, compared to that of the control sample which indicated a decrease in brightness. The samples with cinnamon extract showed a higher a* value compared to the control sample and the sample with ginger extract, which indicates the presence of red color in the cinnamon extract. The b* value was the highest in the sample with ginger extract, the degree of yellowness in other samples was significantly reduced compared to the control sample, which is probably due to the use of cinnamon extract in the formulation, causing the yellow color to be covered by the red color. In general, the highest color change was related to the dessert with 4% cinnamon extract.

**TABLE 6:** Effect of ginger and cinnamon extracts on the color parameter of optimal dessert.

Sample	Formulation	L*	a*	b*	∆E*
1	0% ginger−0% cinnamon extract	90.99 ± 0.00^a^	−2.0230 ± 0.001^a^	14.15 ± 0.02^d^	0.01 ± 0.1^e^
2	0.2% ginger−0.2% cinnamon extract	71.86 ± 0.01^f^	5.0827 ± 0.003^b^	9.27 ± 0.03^c^	20.95 ± 0.01^c^
3	0.1% ginger−0.3% cinnamon extract	71.94 ± 0.01^c^	5.0767 ± 0.002^f^	9.28 ± 0.01^b^	20.91 ± 0.01^f^
4	0.3% ginger−0.1% cinnamon extract	71.87 ± 0.01^d^	5.0967 ± 0.006^c^	9.21 ± 0.00^f^	21.01 ± 0.01^b^
5	0.4% cinnamon extract	66.81 ± 0.01^e^	6.4767 ± 0.002^d^	8.79 ± 0.05^a^	26.2 ± 0.01^a^
6	0.4% ginger extract	84.52 ± 0.01^b^	0.2867 ± 0.001^e^	18.30 ± 0.02^e^	8.02 ± 0.00^d^

*Note*: Different letters denote significant differences (*p* < .05).

### 3.3 Antioxidant properties

The effect of ginger and cinnamon extracts (0.4%) on the antioxidant properties of optimal dessert was investigated (Table 7). According to the obtained results, the inhibition percentage of DPPH free radicals in the control sample was 21.19% and it increased by adding ginger and cinnamon extracts. The antioxidant capacity of ginger extract was significantly higher than that of cinnamon extract as in the samples containing only 0.4% cinnamon or 0.4% ginger, the antioxidant activities were 24.30% and 59.77%, respectively. Also, in the samples containing both extracts, the antioxidant properties rise with increasing the proportion of ginger extract. The IC50 values represent the minimum concentration necessary to inhibit 50% of free radicals. It was 88.30 mg/mL in the control sample and by adding 0.4% of cinnamon and ginger it decreased to 77.04 and 31.94 mg/mL, respectively. It can be seen that the effect of ginger extract in increasing the antioxidant activity of dessert was more than that of cinnamon extract. Ginger is one of the important antioxidant‐rich spices due to having biologically active compounds, such as gingerol, shogoal, and hexahydrocurcumin. Gabbi et al. (2018) produced functional ice cream with high antioxidant properties by using processed ginger. The antioxidant property of cinnamon is related to the presence of phytochemical compounds, such as epicatechin, camphene, eugenol, gamma‐terpinene, phenol, salicylic acid, and tannins. Hamidpour et al. (2015) showed that cinnamon extract has strong antioxidant potential and can be used, instead of artificial antioxidants to reduce lipid oxidation of palm oil and other foods. Considering the higher antioxidant properties of ginger than those of cinnamon, in this research, ginger extract is used as a flavor component with high antioxidant properties in the production of functional dairy desserts.

**TABLE 7:** Antioxidant properties of the different desserts containing ginger and cinnamon extracts.

Sample	Formulation	Antioxidant activity Inhibition of DPPH (%)	IC50 mg/mL
1	0% ginger extract−0% cinnamon extract	21.19^f^ ± 0.98	88.30^f^ ± 0.65
2	0.2% ginger extract−0.2% cinnamon extract	33.61^c^ ± 1.03	54.97^c^ ± 0.71
3	0.1% ginger extract−0.3% cinnamon extract	26.68^d^ ± 1.09	70.55^d^ ± 0.47
4	0.3% ginger extract−0.1% cinnamon extract	40.85^b^ ± 0.97	45.48^b^ ± 0.40
5	0.4% cinnamon extract	24.30^e^ ± 1.12	77.04^e^ ± 049
6	0.4% ginger extract	59.77^a^ ± 0.97	31.94^a^ ± 0.56

*Note*: Different letters on the columns indicate a significant difference (*p* < .05) in Duncan mean comparison test among the different values.

### 3.4 Rheological measurements

#### 3.4.1 Steady shear flow test

The rheological properties of dairy desserts in terms of technological aspects (such as filling), sensory properties (such as texture), and stability are very important. These properties depend on factors, such as the amount of fat, type, and concentration of hydrocolloids (Toker et al., 2013).

**FIGURE 2:**
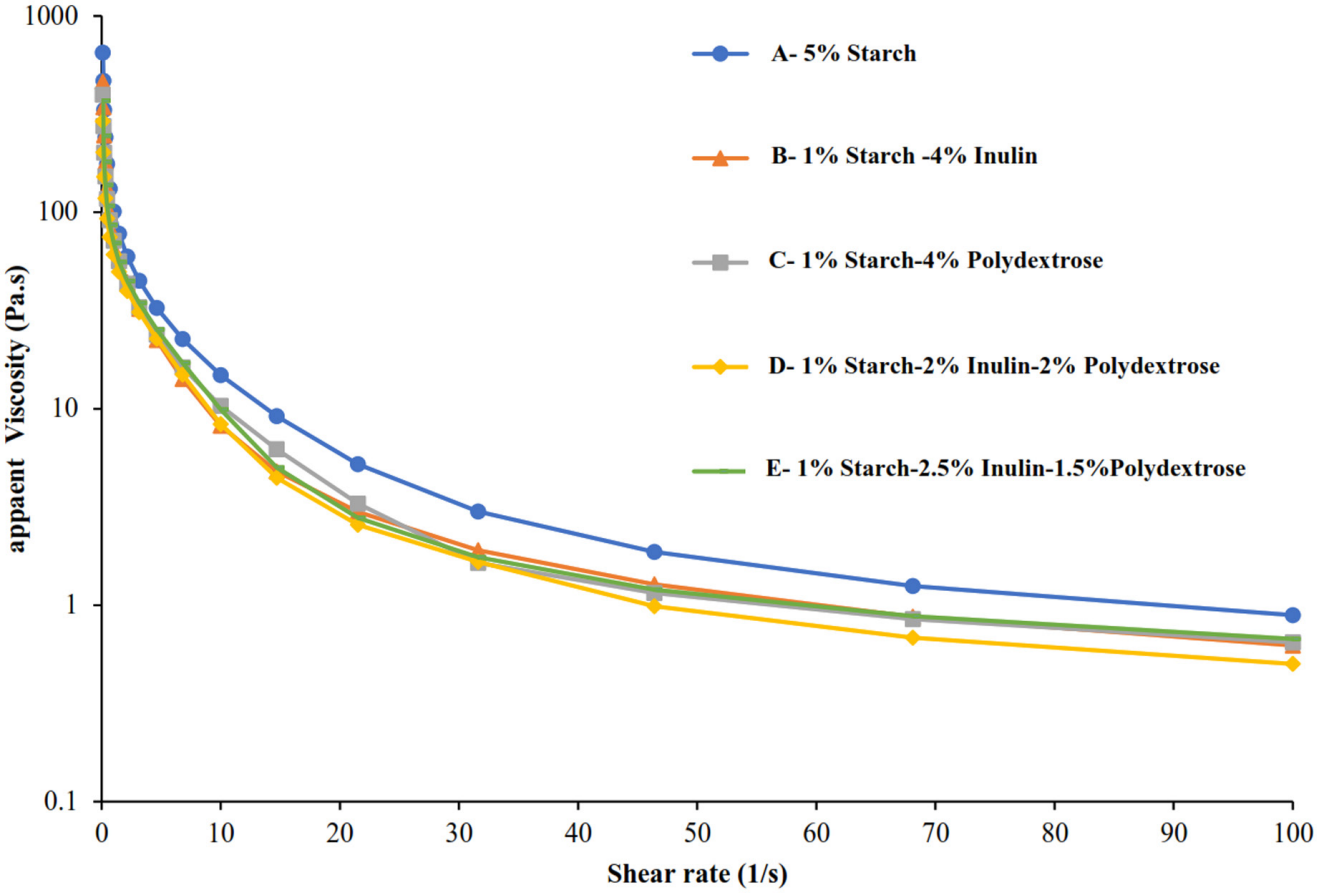
Steady shear flow curves of sample A: 5% Starch, sample B: 1% Starch−4% Inulin, sample C: 1% Starch–4% Polydextrose, sample D: 1% Starch–2% Inulin–2% Polydextrose, and sample E: 1% Starch–‐2.5% Inulin–1.5% Polydextrose.

As can be seen in Figure 2, the apparent viscosity decreased in all samples with the increase of the shear rate, indicating shear‐thinning fluid (pseudoplastic) behavior. As the shear rate increases, the weak bonds are destroyed, which leads to a decrease in viscosity (Gałkowska et al., 2020). Also, fat droplets and protein chains move in the direction of flow, and the flow resistance decreases with an increase in shear strain rate (Balthazar et al., 2017). The apparent viscosity obtained for samples A (5% Starch), B (1% Starch–4% Inulin), C (1% Starch–4% Polydextrose), D (1% Starch–2% Inulin–2% Polydextrose), and E (1% Starch–2.5% Inulin–1.5% Polydextrose) were 651.6, 461.65, 397.35, 291.41, and 370.93 Pa.s, respectively, at the shear rate of 0.01 s^−1^. The apparent viscosity in the control sample was higher than in other samples. The apparent viscosity of the samples containing starch–inulin was higher than the ones containing starch–polydextrose. Also, the lowest viscosity value was observed in the sample containing 2% inulin–2% polydextrose. The chain length of prebiotics affects the technological, rheological, and water retention capacity of prebiotics (Sulejmani et al., 2021; Wang, Wan, et al., 2019; Wang, Yin, et al., 2019). The starch has a higher molecular weight and chain length than inulin and polydextrose, so it has a higher ability to increase viscosity. Inulin has a more linear structure, higher chain length (>23), and higher molecular weight than polydextrose. While polydextrose has a branched structure, higher solubility, and shorter chain length (DP of 12). Therefore, the entanglement of the chains and the absorption of water molecules by inulin are higher than polydextrose, which leads to a higher ability to increase viscosity (Aidoo et al., 2014; Fallourd & Viscione, 2009; Wang, Wan, et al., 2019; Wang, Yin, et al., 2019). Inulin with long‐chain length has better gelling properties and higher viscosity than the short‐chain one (Sulejmani et al., 2021; Wang, Wan, et al., 2019; Wang, Yin, et al., 2019).

#### 3.4.2 Thixotropic behavior (hysteresis loop area)

**FIGURE 3:**
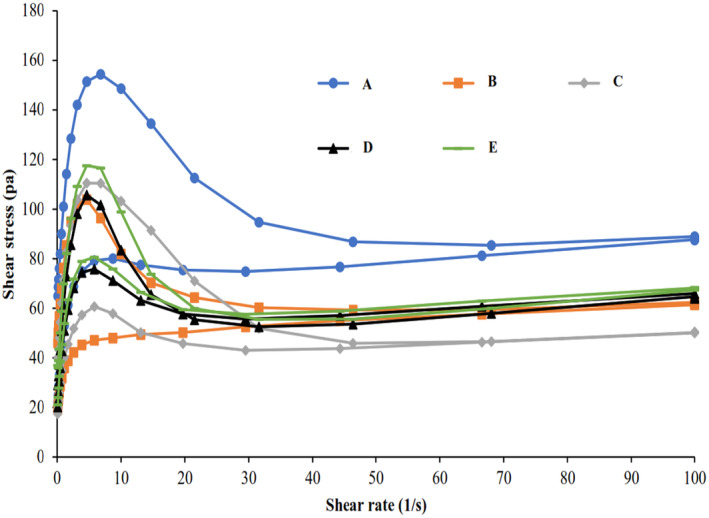
Hysteresis loops of flow curves for sample A: 5% Starch, sample B: 1% Starch−4% Inulin, sample C: 1% Starch–4% Polydextrose, sample D: 1% Starch–2% Inulin–2% Polydextrose, and sample E: 1% Starch–2.5% Inulin–1.5% Polydextrose (25°C).

**FIGURE 4:**
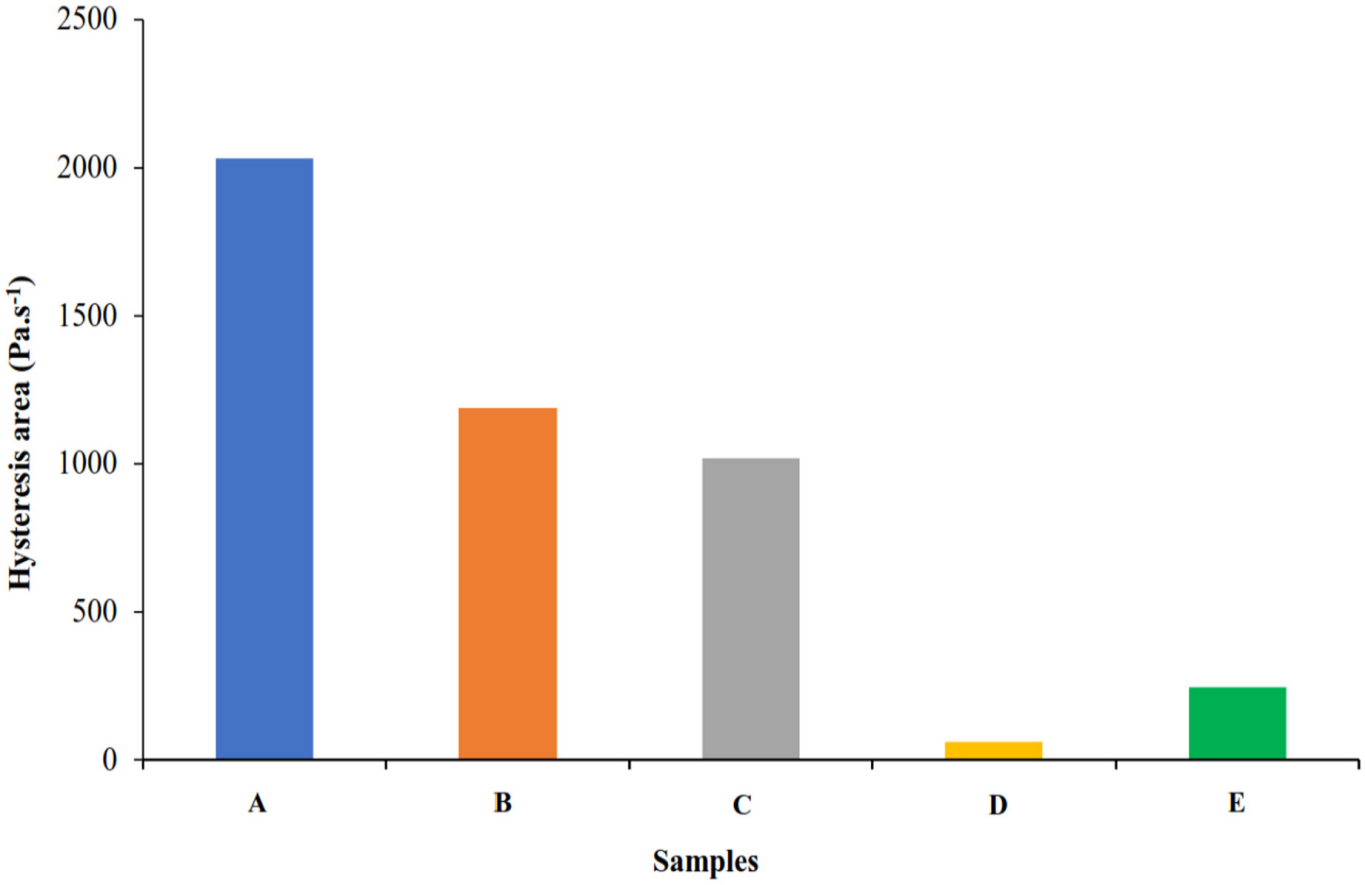
Hysteresis loop area of sample A: 5% Starch, sample B: 1% Starch−4% Inulin, sample C: 1% Starch–4% Polydextrose, sample D: 1% Starch–2% Inulin–2% Polydextrose, and sample E: 1% Starch–2.5% Inulin–1.5% Polydextrose (25°C).

In thixotropic fluids (a type of time‐dependent behavior), apparent viscosity decreases with the increase of shear time at a constant shear rate. One of the methods that are generally used to describe the thixotropic behavior is to determine the area of the upward (ramp up) and downward (ramp down) curves in the shear stress–shear rate diagram, which is called hysteresis. On the other world, the thixotropic degree is usually determined by calculating the area of the hysteresis loop and the larger the area of this loop shows the stronger the thixotropic property and the greater dependence on time (Wang et al., 2016). As shown in Figure 3, in all samples the upward and downward curves did not overlap and a hysteresis loop was observed, so all samples showed thixotropic behavior (Nezhad et al., 2018). The area of the hysteresis loop for samples from 1 to 5 was in the range of 55.83–2030.6, respectively (Figure 4). The control sample showed the strongest thixotropy behavior. In the sample containing only inulin (sample B) compared to the sample containing only polydextrose (sample C), the area of the hysteresis loop was larger and so showed a stronger thixotropic behavior. Similar results were also observed in the research works of Wang, Wan, et al. (2019), Wang, Yin, et al. (2019). The authors showed that adding inulin with different chain lengths at a concentration of 5% to rice starch weakened the thixotropic behavior and reduced the area of the hysteresis loop. Previous studies have shown that the length of the inulin chain plays an important role in thixotropic behavior as with the increase in the length of the inulin chain (7.5%) in dairy desserts, the thixotropic behavior increased and the area of the hysteresis loop became larger (Jorge et al., 2005; Tárrega et al., 2010). Probably, the ability to form a three‐dimensional (3D) network and create a gel‐like state by starch chains is greater than those of inulin and polydextrose due to the higher chain length and molecular weight. Therefore, when the concentration of starch decreases, the formation of a gel network decreases and weakens the thixotropic behavior. Also, inulin and polydextrose have more hydrophilic properties, absorb water at a higher rate, and limit water availability of the starch granules in the medium to swell (Ghanbari et al., 2017).

#### 3.4.3 Dynamic oscillatory shear test

##### Strain sweep analysis

**FIGURE 5:**
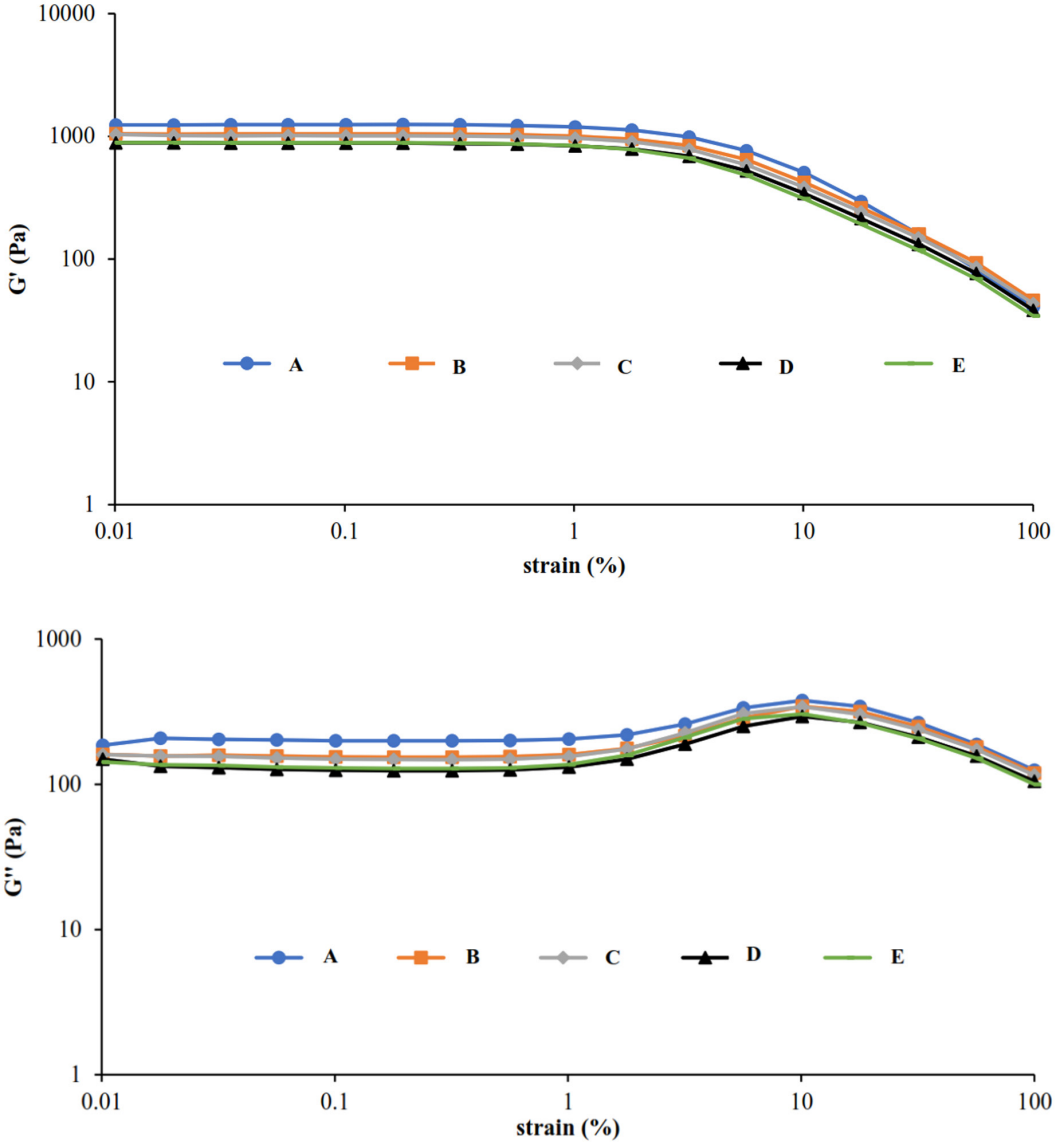
Changes in G′ (filled symbols) and G″ (open symbols) moduli in strain sweep test for sample A: 5% Starch, sample B: 1% Starch −4% Inulin, sample C: 1% Starch−4% Polydextrose, sample D: 1% Starch−2% Inulin−2% Polydextrose, and sample E: 1% Starch−2.5% Inulin−1.5% Polydextrose (*ω* = 1 Hz, 25°C).

The strain sweep test was performed to determine the linear viscoelastic region (LVR) in the strain range of 0.01–100% at a constant angular frequency of 1 Hz and 25°C. In all samples, by increasing the amount of strain amplitude up to almost 10%, the amount of the storage modulus (G') and the loss modulus (G″) were almost constant and G' > G″, which showed the behavior of a weak gel (Figure 5). The values of G' modulus at a strain of 0.01% for samples A (5% Starch), B (1% Starch–4% Inulin), C (1% Starch–4% Polydextrose), D (1% Starch–2% Inulin–2% Polydextrose), and E (1% Starch–2.5% Inulin–1.5% Polydextrose) were 1237, 1051, 1038, 884, and 889 Pa, respectively (Table 7). It can be seen that replacing starch with prebiotics in different concentrations reduced the strength of the gel in desserts. However, the desserts containing inulin showed stronger gel than the ones containing polydextrose. In the amplitude test, strong gels have more LVR region compared to weaker gels (Wang, Wan, et al., 2019; Wang, Yin, et al., 2019). The LVR region in samples containing prebiotics decreased compared to the control sample, indicating a decrease in gel strength. Loss tangent (tag *δ*), which is obtained from the ratio of G“ to G', is one of the parameters describing viscoelastic fluids. In the elastic behavior tan *δ* < 1, and in the viscous behavior tan *δ* > 1 (Hentati et al., 2020). The value of the tan *δ* for a weak gel (biopolymer gels) is between 0.1–1, and it is <0.1 for a really strong gel. As can be seen in Table 8, the tan δ at a strain of 0.01% in samples A to E were 0.15, 0.152, 0.153, 0.168, and 0.160, respectively. Since the tan δ was <1, a weak gel‐like behavior was observed in all samples.

**TABLE 8:** Rheological parameters obtained from strain sweep (frequency = 1 Hz, strain = 0.01%, 25°C).

Sample	Formulation	G′ (Pa)	G″ (Pa)	Tan *δ*
A	5% Starch	1237	185	0.150
B	1% Starch–4% Inulin	1051	160	0.152
C	1% Starch–4% PD	1038	159	0.153
D	1% Starch–2% Inulin–2% PD	884	148	0.168
E	1% Starch–2.5% Inulin–1.5% PD	889	142	0.160

##### Frequency sweep analysis

**FIGURE 6:**
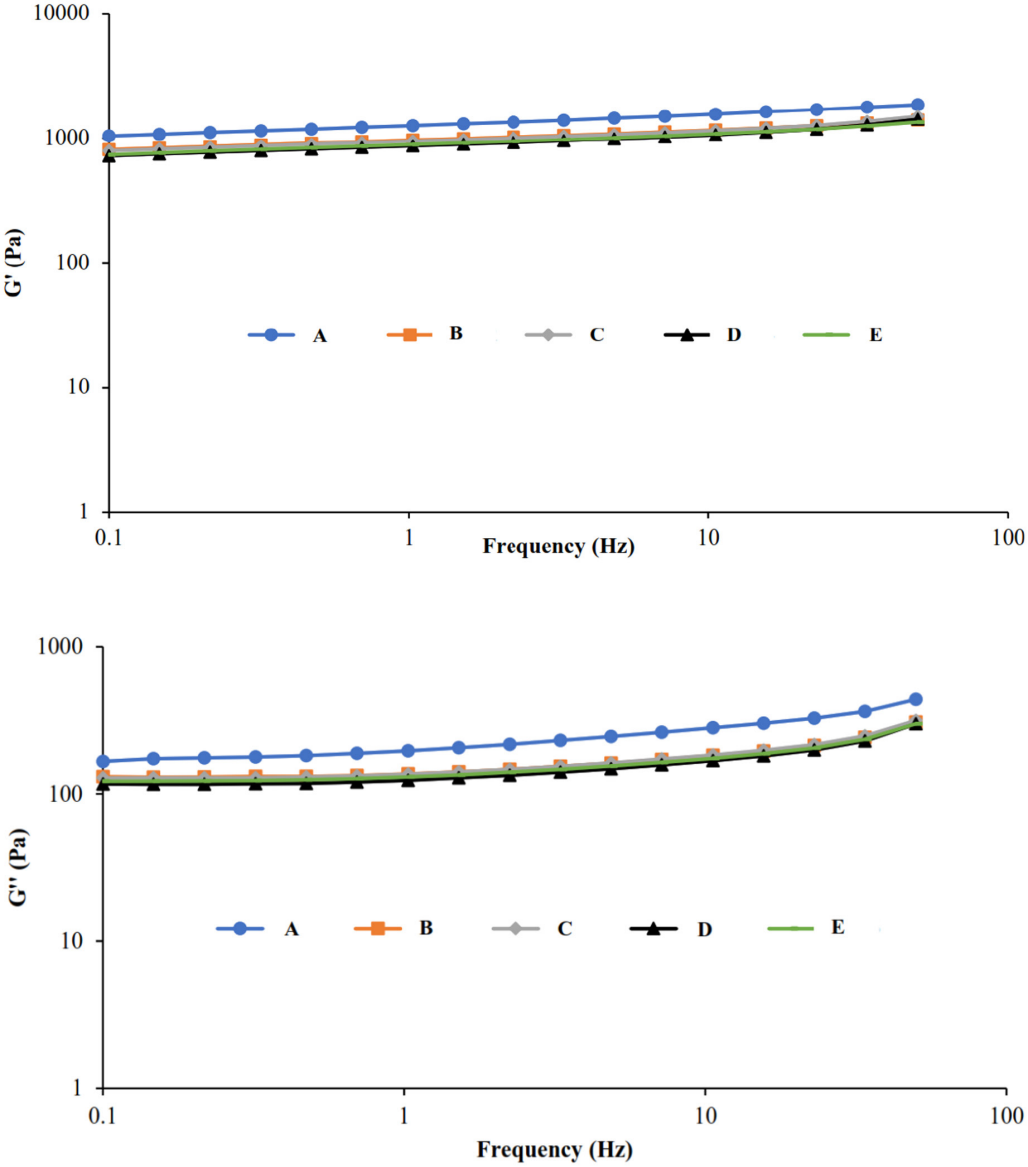
Changes in G′ (filled symbols) and G″ (open symbols) moduli in frequency sweep test for sample A: 5% Starch, sample B: 1% Starch−4% Inulin, sample C: 1% Starch−4% Polydextrose, sample D: 1% Starch−2% Inulin−2% Polydextrose, and sample E: 1% Starch−2.5% Inulin−1.5% Polydextrose (strain = 1%, 25°C).

For all samples, viscoelastic moduli (G′, G′′, and η*) were measured as a function of frequency in the linear viscoelastic region (strain 1%) at the frequency range of 0.1–50 Hz. As the frequency increased, the value of the moduli increased, and G′ > G′′, which indicated a weak gel‐like behavior in all samples (Figure 6). According to the results presented in Table 9, the value of G' modulus for samples A to E was 1040, 809, 792, 723, and 737 Pa, respectively, at the frequency of 0.1 Hz. The value of the G' modulus in the control sample was higher than in the samples containing prebiotics. However, the desserts containing inulin showed a stronger gel than the desserts containing polydextrose. In the research conducted by Wang, Wan, et al. (2019), Wang, Yin, et al. (2019), opposite results were obtained, so the addition of inulin with a high chain length at a concentration of 5% in rice starch increased the value of viscoelastic moduli. The authors reported that inulin–inulin interactions can lead to the formation of an elastic network and lead to an increase in G′. However, similar results were obtained in the research conducted on ice cream containing 10% inulin and polydextrose instead of 10% fat, and by adding a prebiotic, the apparent viscosity and G′ decreased (Balthazar et al., 2017). Also, the samples containing inulin had higher viscosity and elastic modulus than polydextrose. The value of the tag δ for all samples was in the range of 0.158–0.162, which shows that the elastic behavior was dominant in the desserts, and they showed a solid‐like or weak gel behavior. Complex viscosity (η*) is one of the parameters that can be set up in viscoelastic fluids, which expresses the strength of the gel and the total stiffness. As shown in Table 9, the highest η* value was observed in the control sample (1053 Pa.s), and the lowest η* value was measured in sample D (732 Pa.s). It can be concluded that replacing starch with prebiotics reduced the hardness of all desserts.

**TABLE 9:** Rheological parameters obtained from frequency sweep test (frequency = 0.1 Hz, strain = 1%, 25°C).

Sample	G′ (Pa)	G″ (Pa)	Tan *δ*	η* (Pa.s)
A	1040	165	0.158	1053
B	809	131	0.161	819
C	792	128	0.161	802
D	723	117	0.161	732
E	737	122	0.162	747

## 4 CONCLUSION

In this research, a practical formula for a low‐calorie, high‐antioxidant prebiotic dairy dessert was presented using low‐calorie sweetener, ginger and cinnamon extracts, and replacement of starch with prebiotic fibers (inulin and polydextrose). Despite the many challenges in the formulation of new products, the Design Expert software and the D‐optimal mixture design method were very efficient in formulating functional desserts. Although the full replacement of starch with prebiotics caused an increase in syneresis and a decrease in viscosity and thixotropic behavior, optimization results showed that starch in dairy desserts can be replaced to a great extent with inulin and polydextrose without unacceptable organoleptic and syneresis changes. The final optimal formula included 1% starch, 2.49% inulin, and 1.51% polydextrose. The highest antioxidant property was related to the dessert containing 0.4% ginger extract. This formulation can be used in any dairy dessert based on starch to produce a functional product.

## AUTHOR CONTRIBUTIONS

**Seyyedeh Leila Hosseinipour:** Investigation (equal); writing – original draft (equal). **Babak Ghanbarzadeh:** Project administration (equal); supervision (equal); writing – review and editing (equal). **Vahid Mofid:** Software (equal). **Mostafa Soltani:** Validation (equal). **Hedayat Hosseini:** Validation (equal).

## FUNDING INFORMATION

There has been no significant financial support for this work that could have influenced its outcome.

## CONFLICT OF INTEREST STATEMENT

We wish to confirm that there are no known conflicts of interest associated with this publication.

## Data Availability

The data that support the findings of this study are available on request from the corresponding author. The data are not publicly available due to privacy or ethical restrictions.
